# Novel optimized biopolymer-based nanoparticles for nose-to-brain delivery in the treatment of depressive diseases †

**DOI:** 10.1039/d0ra04212a

**Published:** 2020-08-04

**Authors:** Alessandro Sorrentino, Antonino Cataldo, Riccardo Curatolo, Pietro Tagliatesta, Luciana Mosca, Stefano Bellucci

**Affiliations:** INFN-Laboratori Nazionali di Frascati Frascati Rome Italy bellucci@lnf.infn.it; Raithor Srl Rome Italy; Dipartimento di Scienze e Tecnologie Chimiche, Universita' di Roma Tor Vergata Rome Italy; Department of Engineering, Polytechnic of Marche University of Ancona Ancona Italy; Department of Biochemical Sciences, Sapienza University of Rome Rome Italy

## Abstract

A valid option to bypass the obstacle represented by the blood–brain barrier (BBB) in brain delivery is the use of the unconventional intranasal route of administration. The treatment of depressive diseases, resulting from the depletion of a neurotransmitter in the inter-synaptic space, such as serotonin, is indirectly treated using molecules that can permeate the BBB unlike the latter. In the present article, a set of nanovectors were produced using a mucoadhesive biopolymer, *i.e.* alginate (Alg). Optimizing the reaction, polymeric nanoparticles having diameter of 30–70 nm were produced, and water stable multi-walled carbon nanotubes functionalized (MWCNT-COOH)/Alg complexes were obtained. These nanovectors were loaded with serotonin, evaluating drug loading/release. By means of Raman microscopy, the cellular internalization of the (MWCNT-COOH)/Alg complex was demonstrated. A complete biocompatibility on neuronal cells was proved for the whole set of nanovectors. Finally, a method of self-administration was tested, which involves the use of a household apparatus, such as an aerosol machine, observing a fine particulate, able to deliver the nanovectors through the nose.

## Abstract 2

A valid option to bypass the obstacle represented by the blood–brain barrier (BBB) in brain delivery is the use of the unconventional intranasal route of administration.

## Graphical abstract

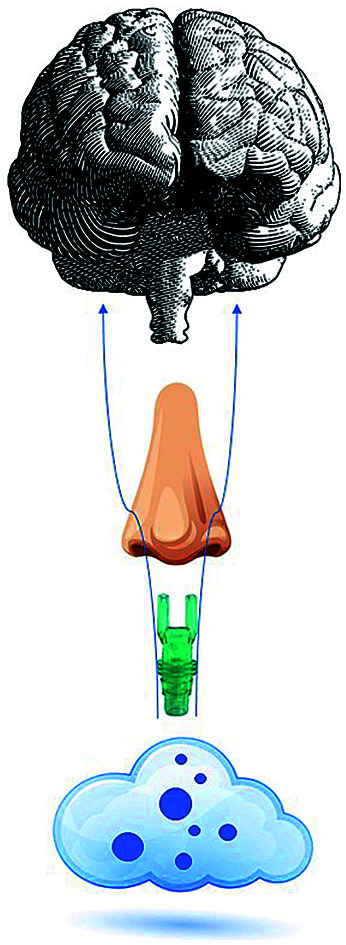

## 1 Introduction

The possibility to target the brain through the nose has attracted a wide interest in the scientific community. In fact, the number of papers published every year about drug delivery to the brain through the nose (NBD) is undergoing an exponential growth. The NBD has been demonstrated in animals that have a decidedly more developed olfactory epithelium than humans.^1,2^ The paper dealt with the synthesis of new drug delivery systems able to reach the brain through nose to brain delivery.

The drug delivery to the brain is hindered by the non-permeability of blood brain barrier (BBB) by many molecules including drugs.^3^ This mechanism is a defence of the human body from exogenous molecules that could damage the brain, altering its normal functions.^3,4^ Serotonin, (an endogenous neurotransmitter), is one of the molecules stopped by BBB ad a low concentration of it, in the intersynaptic space, is responsible for neurological problems, such as depression.^5–7^

Since BBB hampers the direct oral administration of the drug, the treatment of these pathologies occurs indirectly through the administration of SSRI (serotonin selective reuptake inhibitor), such as paroxetine. This molecule gives a good results for the treatment of depression disease but it shows a non-negligible affinity to bind muscarinic receptors, unchaining a series of side effects^8^ often not tolerated by the patient who is forced to stop the treatment.

The intranasal administration would allow for direct administration and brain delivery of serotonin in humans, by avoiding the passage through the BBB.^4^

It is well known that the respiratory epithelium is formed by a series of supporting cells that serve to give a mechanical support to the bipolar neurons. These neurons are formed by non-mobile cilia that are confined in the nasal cavity and going upwards, become thinner in an unmyelinated axon to form beams of axons in the lamina propria, that exceed the cribriform plate passing through holes. There are about 1500 olfactory receptors on bipolar sensory neurons, converging on a mitral cell or a quilted cell in the olfactory bulb. The projections go to the amygdala, the prepiriform cortex and the entorhinal cortex as well as in the hippocampus, hypothalamus and thalamus.^9–11^

The passage to the brain could be possible using nanovectors^12^ and it can take place mainly by two mechanisms and more precisely exploiting (i) the olfactory and trigeminal nerve pathways in the nasal cavity (*i.e.*, the so-called “axonal transport”) as well as by (ii) paracellular transport across the spaces between cells or (iii) by transcellular transport across the basal epithelial cells (*i.e.*, the epithelial pathway).^13,14^ The transcellular transport is usually favoured for lipophilic molecules or nanovectors while the paracellular transport is usually favoured for hydrophilic molecules or nanovectors.

Starting from this knowledge and given the great interest in this research environment the wide use of chitosan in the literature, encouraged the idea of generating an alternative set of polymeric nanovectors based on another muco-adhesive biopolymer such as the alginate.^15–21^

The aim of the work is to prepare nanovectors that have different chemical–physical characteristics so that the three possible ways to reach the brain through the nose can be covered.

To investigate the first and third routes, MWCNT/alginate complex was selected, due to their physical–chemical characteristics as lipophilicity. The CNT therapeutic experiments, for the majority in the preclinical stage, started no more than 20 years ago: nevertheless, many attempts have been carried out in gene peptide and drug delivery^22–27^ for many applications, such as cancer, lymphatic, ocular and brain targeting. About brain targeting, many results have been reached, such as the reactivation of neuronal pathway after brain injury,^28,29^ and the delivery of drugs^22,30^ passing through the BBB. Even though the BBB passing was demonstrated by the gastrointestinal way, a potential uptake of pristine MWCNT from other organs through the blood torrent could occur, inducing side effects or toxicity in other not targeted organs. To reduce the undesired not brain uptake, without specific functionalization,^31^ the nose to brain approach to distribute MWCNT was used.

Nanospheres and nanoaggregates of alginate have been prepared, for the second route as polar nanovector.

The use of alginate can increase the retention time in the nasal cavity by means of muco-adhesive properties and decrease the absorption by the respiratory epithelium by means of the hydrodynamic volume.

The muco-adhesive properties hinder the mucociliary clearance, decreasing the expulsion towards the nasopharynx; the absorption usually occurs for small molecules (<1000 Da): increasing the hydrodynamic volume of the drug, the uptake decreases.

The generated nanovectors were loaded with the drug (serotonin or 5-hydroxytryptophan) and then cellular toxicity tested at 24 h and 48 h on SHSY-5Y cells, in order to verify a possible toxicity towards brain cells.

For selected samples, the release kinetics were also studied, as the goal is to obtain a slow release, given the target pathologies that we have chosen to treat. Treatment of depression involves the slow reaching of the stationary state of the drug in the organ, to avoid the inducing of mood swings in the patient.

The internalization of the nanovectors engineered to travel along the axonal path was also studied on neuronal cells, through Raman mapping in the *XY* plane and series in *Z*.

Finally, the method of administration was studied, allowing us to obtain a fine particulate by aerosol, thus favouring the self-administration of the system.^32^

## 2 Experimental part

### 2.1 Synthesis of nanospheres

**Fig. 1:**
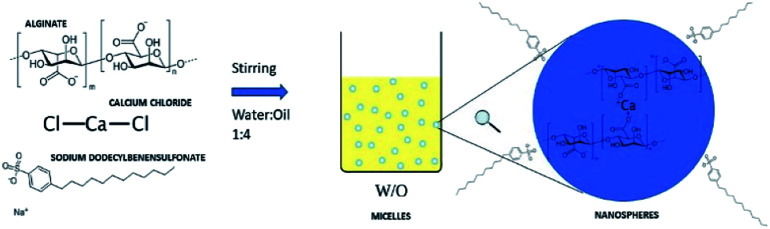
Schematic representation of the synthesis of nanospheres from the gelation reaction in emulsion.

The synthesis of nanospheres was carried out according to the alginate gelation in the presence of calcium ions (Fig. 1). The water/oil ratio used is 1 : 4. In order to eliminate completely the oil phase in post process step, the oil phase was formed by dichloromethane, having low boiling point (39.6 °C). The anionic surfactant sodium dodecylbenzenesulfonate (SDBS, Sigma-Aldrich) was used to avoid ionic interactions with the drug.^33–37^

The first step involves a screening on surfactant concentrations. The ratio 1 : 1 equivalent of alginate (*E*_Alg_) : Ca^2+^ was used. Sodium alginate was purchased from Sigma-Aldrich and CaCl_2_ from Carlo Erba reagents.

The second step was the optimization of the ratio (*E*_Alg_) : Ca^2+^ in the ratio 1 : 3 and 3 : 1. All the tests were carried out using magnetic stirring at 3000 rpm.

### 2.2 Synthesis of nanoaggregates

**Fig. 2:**
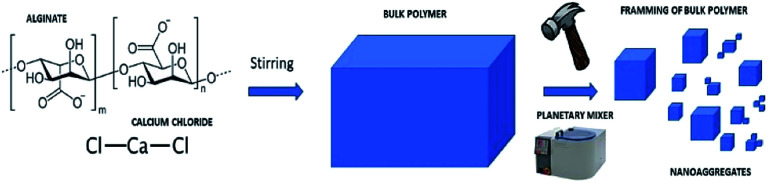
Schematic representation of the synthesis of nanoaggregates by top down approach.

The synthesis of the nanoaggregates envisages exploiting a top-down approach. Gelation of alginate in water was obtained at a concentration of 0.01 M ratio 1 : 1 (*E*_Alg_) : Ca^2+^. The next step was the disintegration of the polymer bulk using different systems including ultrasonic tip Sonics Vibra-cell (40 MHz, 20 min), IKA T-10 basic Ultra Turrax (level 3, 20 min) and planetary mixer Thinky ARV-310 (2000 rpm 8 min) (Fig. 2).

### 2.3 Preparation of MWCNT-alginate system

**Fig. 3:**
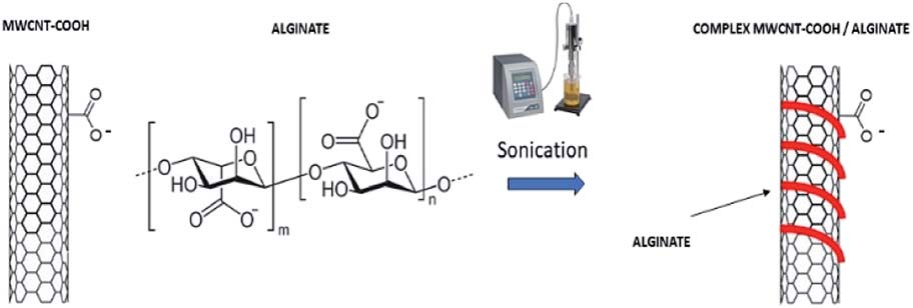
Schematic representation of the synthesis of MWCNT-COOH/Alg complex.

Multi walled carbon nanotubes, functionalized with carboxyl groups (MWCNTs-COOH), were dispersed in a water solution at a concentration of 0.5 mg mL^−1^ and alginate was subsequently added in different ratios (w/w) 10 : 1, 1 : 1, 1 : 10 MWCNT-COOH : alginate. The solution was exposed to ultrasounds using an ultrasonic tip (40 MHz, 1 s on/off, 40 min) to facilitate wrapping of CNTs (Fig. 3).

### 2.4 Characterization of generated nanovectors

The particle size and surface morphology were analysed using a scanning electron microscope (SEM) Tescan Vega 3. The electron beam energy ranged between 10–30 kV. To analyse the chemical composition, spot elemental analysis was performed with energy dispersive X-ray spectroscopy (EDS) using a Quantax Bruker detector.

The internalization and the interaction drug/carrier were analysed using Raman spectroscopy Renishaw inVia equipped with a green laser (532 nm).

### 2.5 Drug loading

The 5-hydroxytryptophan and serotonin were added directly during the synthesis of the nanovectors in the aqueous phase at a concentration of 10^−2^ M. Then, the vectors were centrifuged at 12 000 rpm, 4 °C for 450 min, the supernatant was eliminated and the precipitate dried.

The samples containing nanotubes were characterized by Raman spectroscopy using green laser (532 nm) with a lattice of 1800 L mm^−1^ and the samples containing alginate nanospheres and nanoaggregates were characterized by ATR-FTIR spectroscopy using Agilent technologies Cary 630 FTIR.

### 2.6 Drug release

To gain information on the drug release kinetic the centrifuged samples were placed in an aqueous solution of phosphate buffer 10 mM at 36 °C with slow stirring and aliquots of 1 mL were taken. The concentration of the analyte was subsequently obtained by UV spectroscopy at an absorption max of 275 nm. The samples were analysed by Varian Cary 50 spectrophotometer and the spectra were smoothed by Savitzky–Golay at 30 pt. Concentrations were calculated from the max absorbance values by the Lambert–Beer equation. The values were corrected, and subsequently a decreasing mono-exponential fit was carried out to assess the release kinetic.

### 2.7 Cytotoxicity evaluation

SH-SY5Y neuroblastoma cells were grown in DMEM/F-12 medium containing 10% fetal bovine serum (Gibco BRL Life Technologies Inc., Grand Island, NY, USA) and 2 mM l-glutamine at 37 °C in a humidified atmosphere with 5% CO_2_. 15 000 cells per well were seeded in 100 μL of DMEM/F12 in 96 well plates. The cells were incubated for 24 h to allow seeding and then an aliquot of 20 μL per well of unloaded and loaded nanovectors, at a chosen concentration, were added to the wells. Untreated cells were used as control. The cells were incubated in the presence of the vector up to 48 h, then 25 μL of 5 mg mL^−1^ thiazolyl blue tetrazolium bromide (MTT) in phosphate buffered saline (PBS) was added per well and then incubated again for 2 h. The supernatant was carefully removed, and the formazan salts dissolved in 100 μL per well of DMSO. Optical density of each well was determined at 570 nm with a reference at 690 nm using Appliskan microplate reader, Thermo Scientific.

### 2.8 Internalization of loaded nanovectors

For the MWCNT-COOH/alginate and nanoaggregates sample, the internalization was evaluated following the treatment of a 10 μg mL^−1^ solution of the sample on a SHSY-5Y cell line by Raman spectroscopy (*λ* = 532 nm). The Raman characterization was carried out after 5 h of incubation directly in the plates, using single spectrum and mapping setup.

### 2.9 Aerosol type deposition

A 5 mg mL^−1^ nanovectors solution was placed in an aerosol fork and attached to a pressure generator set at 1.3 bar. Then it was deposited for 10 s on an aluminium coated slide 45° curved (Fig. 15), to avoid fluorescence in the following Raman investigations, able to reconstruct the size of droplets.

## 3 Results and discussion

### 3.1 Characterization of alginate nanoparticles and drug loading

**Fig. 4:**
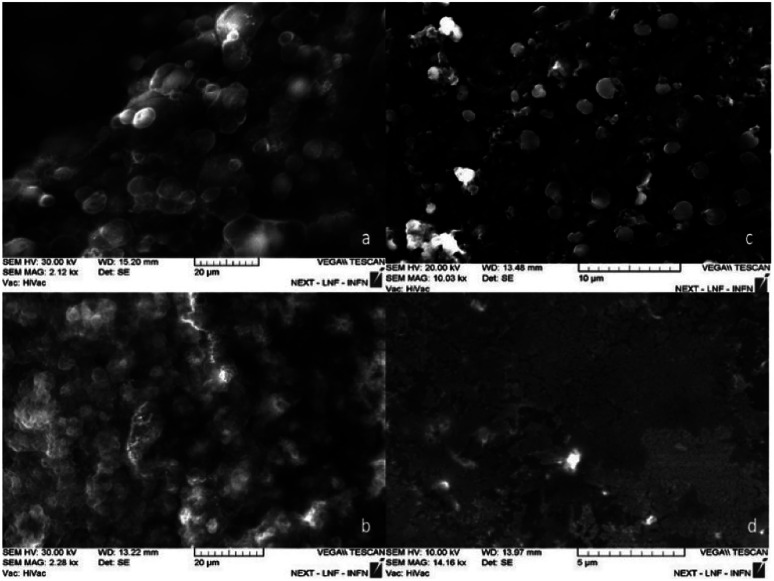
It is possible to see the variation in particle size of the particles obtained when the surfactant concentration varies. In figure (a) 2.5 10^−2^ M to (b) 10^−2^ M to (c) 5 10^−3^ M and (d) 10^−3^ M.

The nanospheres obtained show a diameter of 220 ± 4 nm. The SEM micrographs in Fig. 4 shows the influence of the surfactant concentration on the particles size: decreasing the surfactant concentration, the particles size decreases. At lower concentration, it is possible to notice the presence of spheres with diameter smaller than 30 nm (Fig. 4d). This results are attributed to the fact that an increase in surfactant concentration leads to an enlargement of the aqueous pool with an increase in the size of the droplets.^38^

Too small particles could be too reactive in the nasal cavity, and this fact might be bad for a slow release. In this regard, we decided to investigate the synthesis at a concentration of 5 10^−3^ M surfactant by varying the ratio (*E*_Alg_) : Ca^2+^ ratio 1 : 3, 3 : 1. As the LaMer diagram^39^ suggests, an increase in the Ca^2+^ concentration leads the system to the supersaturation zone, with a consequent increasing in nucleation times which results in a polydisperse system with large spheres.^40^

Decreasing the concentration of Ca^2+^, the system goes down towards the short nucleation zone (in LaMer diagram) that allows to observe a monodisperse system (Fig. 1Sa†).

The 1 : 1000 (V/V) dilution test performed at the lowest calcium concentration shows the presence of monodisperse spheres with a diameter of 220 ± 4 nm (Fig. 1Sb†).

**Fig. 5:**
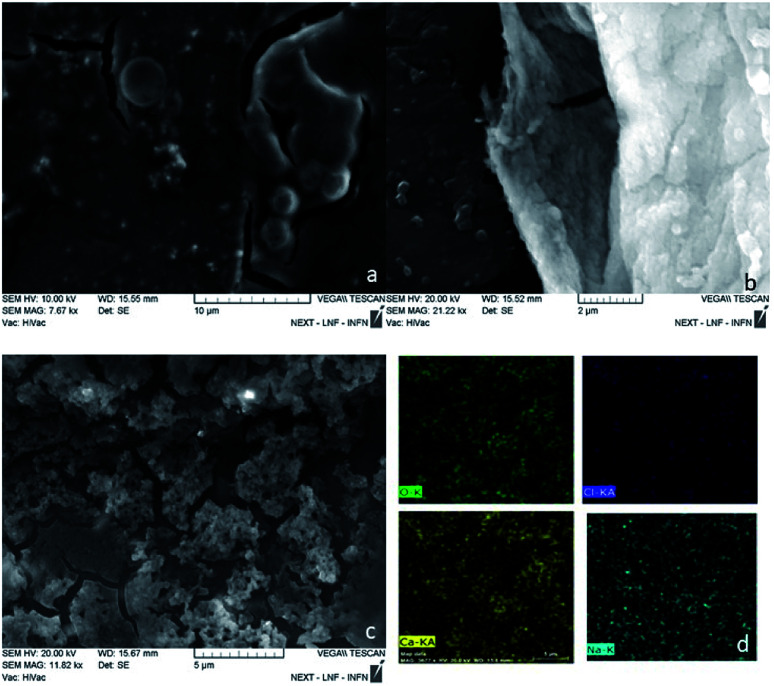
Effects of different top down setup on particle morphology the results obtained with (a) ultrasound tip, (b) homogenizer and (c) planetary mixer. In (d) the EDX mapping of panel (c) are reported on O–K (green), Cl-Kα (blue), Ca-Kα (yellow), Na–K (light blue).

Applying the top down approach, the polymer bulk was broken down using ultrasonic and shear forces (Fig. 5). The results with ultrasonic tip show the presence of micrometric spheres (2–3 μm). The process was performed with U-Turrax homogenizer showing the presence of irregular aggregates with size around 400 nm (Fig. 5b). Test carried out in an ARV-310 planetary mixer shows the presence of small aggregates (Fig. 5c). EDS measurements were made on the material to discriminate from the presence of salt crystals (Fig. 5d). Once the presence of polymer was confirmed, a 1 : 1000 (V/V) dilution test was performed, revealing particles having 30–70 nm diameter size (Fig. 2S†).

Given this excellent result, together with the speed of preparation, the ARV-310 setup was chosen for in-depth tests.

**Fig. 6:**
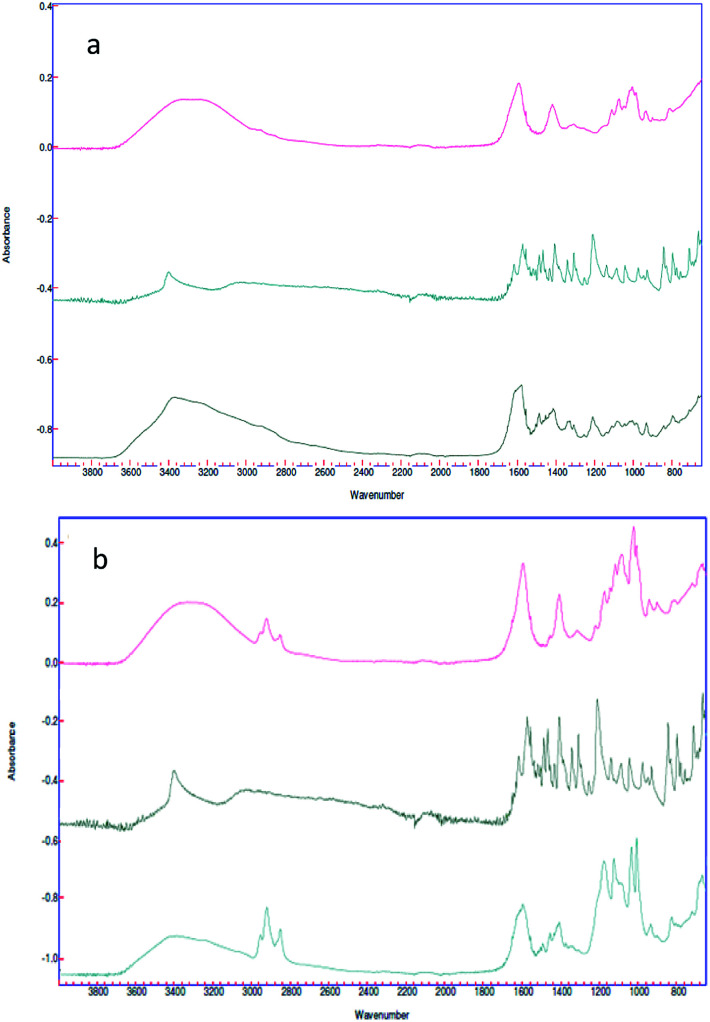
(a) Spectra of the alginate nano-aggregates (red), 5-HTP (green) and complex (black); (b) spectra of the alginate nanosphere complex (red), the 5-HTP complex (black) and the complex (green).

In spite of the fact that customary characterizations employed in the literature to confirm the drug loading are Thermo Gravimetric Analysis (TGA)^41^ and High Performance Liquid Chromatography (HPLC),^42^ we make use in this work of ATR-FTIR spectroscopy, in a way analogous to earlier works. Indeed, in Bhalekar *et al.*,^43^ FTIR spectral peaks where used to show the drug entrapment in lipid nanoparticle matrix, whereas in Madni *et al.*,^44^ ATR-FTIR spectroscopy was employed to investigate the possible intermolecular interaction of cytarabine with deacetylated chitosan and tripolyphosphate in the resulting physical blends and crosslinked nanoparticulate system. In the same spirit, in the present work, ATR-FTIR spectroscopy (Fig. 6) was carried out for each sample engineered for the paracellular pathway.

Spectra of empty nanovectors, 5-HTP and nanovectors loaded with 5-HTP were recorded.

In both cases the spectrum of the loaded nanovectors is given by the convolution of the two different spectra. It is also possible to see, in the spectrum of the loaded sample, the presence of the typical signals of the amino function of the drug is highlighted, such as N–H stretching *ν* = 3400 cm^−1^, C–N stretching *ν* = 2200 cm^−1^ and N–H bending *ν* = 1200 cm^−1^ (Fig. 6a black spectrum and Fig. 6b green spectrum).

### 3.2 Characterization of MWCNT-alginate and drug loading

MWCNT-COOH (low stability in aqueous solution) have been stabilized in water with alginate by hydrogen bonding interactions and mechanics promoted by ultrasonic tip mixing.^45^ Several ratios have been tested and allowed to stand for 30 days at room temperature and subsequently observed (Fig. 3Sa†). The sample with the highest concentration of alginate did not show stability, while the other two appeared to be equally stable. Such samples having the lowest concentration of biopolymer were more stable. SEM investigations were carried out to control the results. When the concentration of surfactant increased, increasingly larger polymer aggregates/MWCNTs were formed, which were too heavy to remain in solution and precipitated (Fig. 3Sd†).

The presence of nanometric spheres (Fig. 3Sb and c†) was attributed to the presence of bivalent cations used in CVD growth processes not properly purified by the nanomaterial, which promoted nucleation during the cavitation process of the ultrasonic tip.

**Fig. 7:**
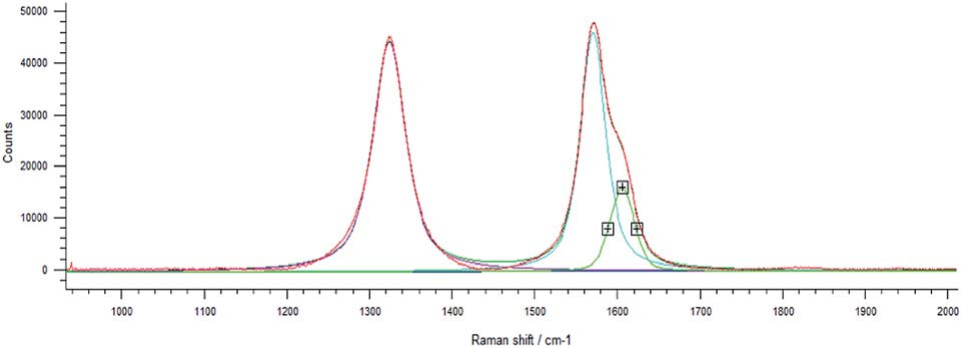
Raman spectrum at high resolution shows D, G and D^I^ peaks.

Drug loading obtained directly in synthesis was observed by Raman spectroscopy for MWCNT-COOH/alginate nanovectors. Due to the very low Raman scattering properties of alginate and drugs, the MWCNTs signals overwhelm the signals of the other constituents (Fig. 7). In addition to the information about the drug loading provided by ATR-FTIR spectroscopy, which is analogous to the previously illustrated case of drug on alginate nanoparticles, without the presence of nanotubes, here we decided to analyse the Raman features, as a way to further determine the drug loading and interactions. In Saupe *et al.*,^46^ Raman spectroscopy was used to investigate the structural changes induced by loadings in complex systems. Raman scattering was utilized in Lim *et al.*^47^ to characterize the dopamine loading on chitosan–gold nano composites. In the present work, we observe relevant changes (blue shifts) in the Raman feature of our systems, as way (additional to ATR-FTIR) to further confirm the effective loading using the MWCNT-COOH as probe. In this regard, the displacement of G, D and D^I^ bands towards larger wave numbers was observed.

This phenomenon is called blue shift and occurs when the polarizability of the molecules is altered by interactions with exogenous molecules.

The analysis was carried out for the MWCNT-COOH/alginate 10 : 1 sample, the lowest concentration of stabilizer. The drug, 5-HTP, was also added, favouring the π–π interaction. The experiment with serotonin was not repeated.^48^ It could be seen that, upon the addition of alginate (Table 1), a 4.4 cm^−1^ shift of the G band occurs, which increases by an additional 2.7 cm^−1^ shift when the drug is added to the system.

**Table 1:** Raman shift of the D, G and D^I^ peaks

Curve name	MWCNT	MWCNT/Alg	MWCNT/Alg/drug
D band	1323.9 cm^−1^	1343.8 cm^−1^	1345.6 cm^−1^
G band	1571.3 cm^−1^	1575.7 cm^−1^	1578.4 cm^−1^
D^I^ band	1606.5 cm^−1^	1612.5 cm^−1^	1615.5 cm^−1^

### 3.3 Drug release

**Fig. 8:**
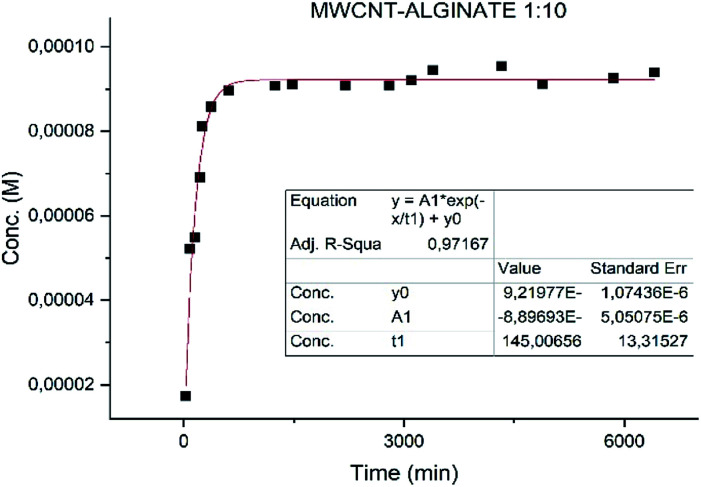
Fit of the release of MWCNT-COOH/Alg/drug sample.

**Fig. 9:**
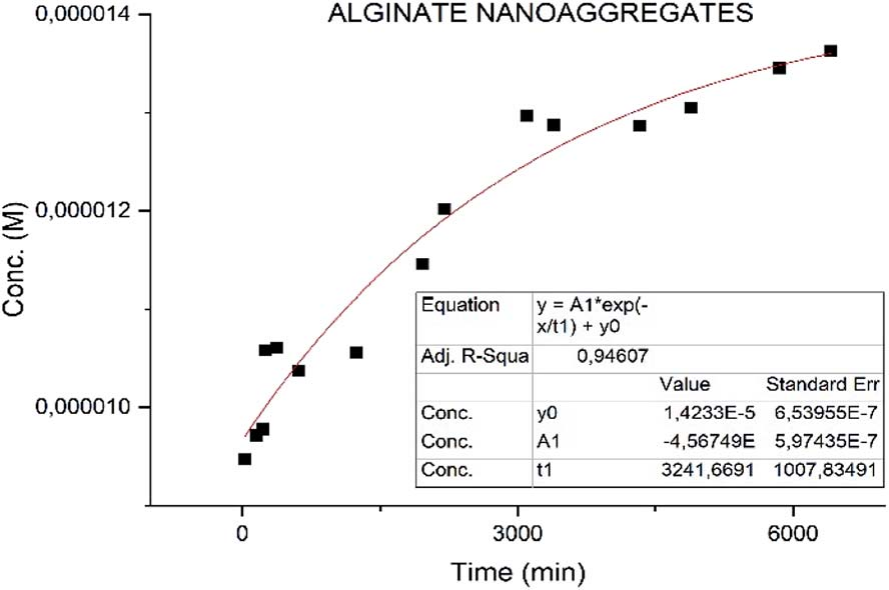
Fit of the release of nanoaggregates/drug complex.

Different results were obtained for the samples. Specifically the sample MWCNT-COOH/Alg has a faster release with *t*_1/2_ of 2 h (Fig. 8) and much slower for the alginate nanoaggregates with *t*_1/2_ 36 h (Fig. 9).^49,50^

Particular attention is deserved by the nano-aggregate alginate sample. The latter shows an extremely slow release, excellent for the treatment of depressive diseases (Fig. 9).

**Fig. 10:**
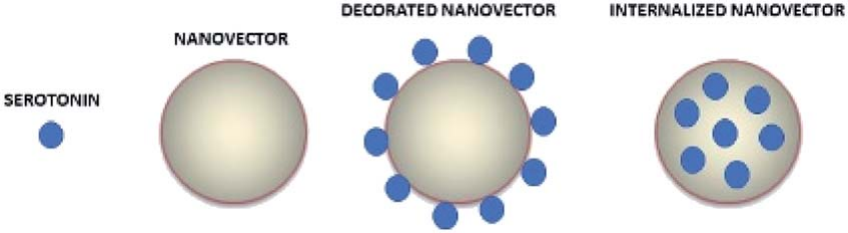
Schematic representation of drug positioning in the vector.

These results could lead to a determination of how the neurotransmitter is positioned in the vector. In particular in a situation of fast release (MWCNT-COOH/Alg/drug), with an initial burst phase, the vectors are probably decorated with the neurotransmitter, while in the case of slow release (nanoaggregates) the drug could be inside the vectors as shown in Fig. 10. These results are preliminary, will need to be confirmed by further investigation.

### 3.4 *In vitro* cytotoxicity

**Fig. 11:**
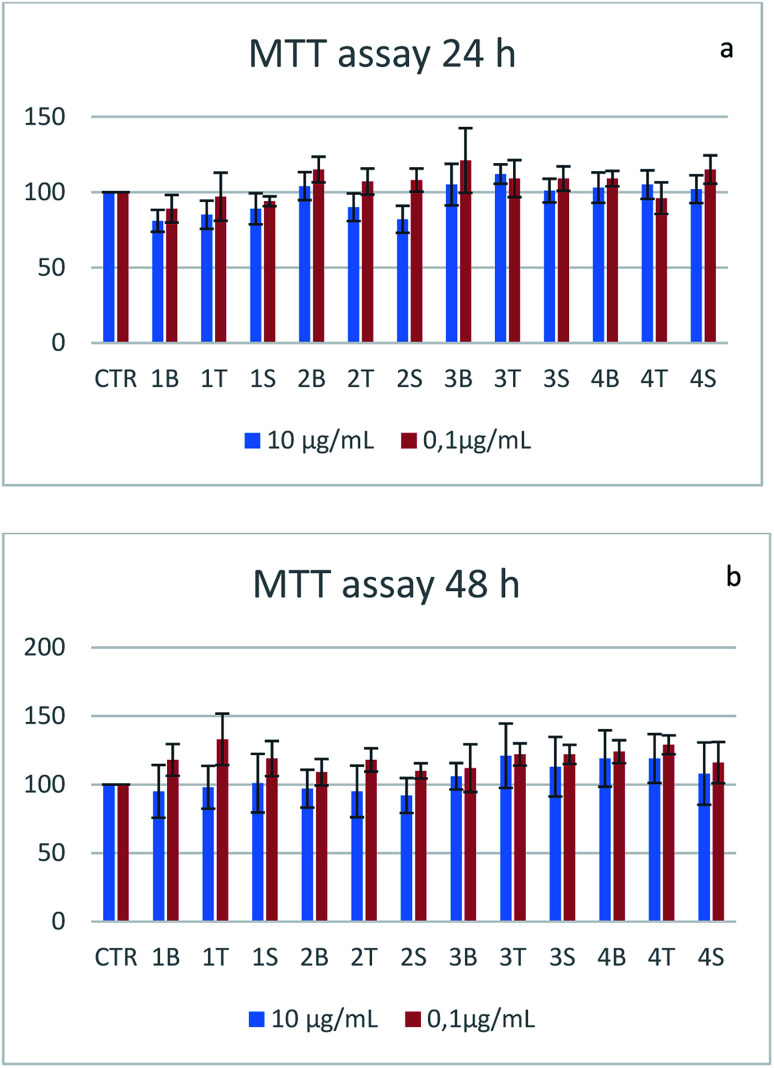
MTT assays at 24 h (a) and 48 h (b). The samples are identified with a number (1 for MWCNT–Alg–drug 10 : 1 ratio 2 for MWCNT–Alg–drug 1 : 1 ratio, 3 nanoaggregates–drug complex, 4 for nanospheres–drug complex) and a letter (B for the samples without drug, S for the samples charged with serotonin and T for the samples charged with 5-HTP).

Cell toxicity was assessed on the SHSY-5Y cell line. Cells seeded in 96-well plates are treated with the empty vector or vectors loaded with the two drugs (5-HTP, serotonin) at two different concentrations and cell viability is tested at 24 h and 48 h by means of the MTT assay. At both tested concentrations the samples are non-toxic (Fig. 11). Cell viability shows a decrease when the samples are treated with nanotubes, although the value is still above the toxicity threshold (80%). The alginate-based samples show an increase in cell viability that could be probably due to the metabolization of the biopolymer.^51^

### 3.5 Internalization

Usually, the cellular internalization studies have been carried out by means of confocal fluorescence microscopy. This technique required the use of fluorescent markers, or genetic engineering.^52^ The confocal Raman spectroscopy could be a non-invasive technique, which does not require any cellular manipulation.^53–55^ Moreover, although this technique was useful for cellular *in vitro* studies, to the best of our knowledge, it has been used to characterize cancer cells (pancreatic, breast, cervical tumours)^53^ and not yet neuronal ones. SHSY-5Y cells were treated with the MWCNT-COOH/Alg 10 : 1 sample, which is the engineered carrier to travel the first and third routes. The cells were exposed to the samples for 5 h and then washed and fixed on the plate. Using a green laser (532 nm), the cells were identified through the optical microscope and subsequently mapped in *XY*, with a step of 0.2 μm, on the peak D of the MWCNT-COOH. We chose to analyse the D peak because of the presence of a large signal coming from the matrix at a Raman shift similar to the G band Raman shift. To avoid a false positive test, we selected the D band as a pointer to detect the MWCTN-COOH/Alg complex.

**Fig. 12:**
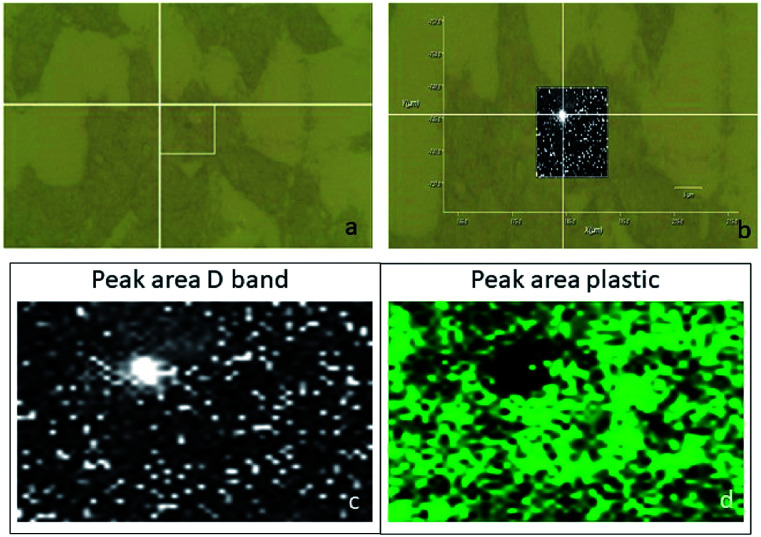
(a) Optical image of the analysed cell; (b) superimposition of the mapping results of the D peak area (c) on the optical image; mapping results of the plastic peak area ( d).

**Fig. 13:**
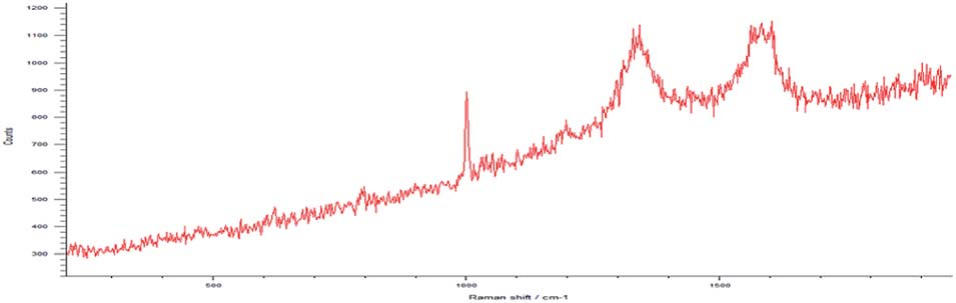
Raman point spectrum in the position where the MWCNT-COOH/Alg complex is present.

**Fig. 14:**
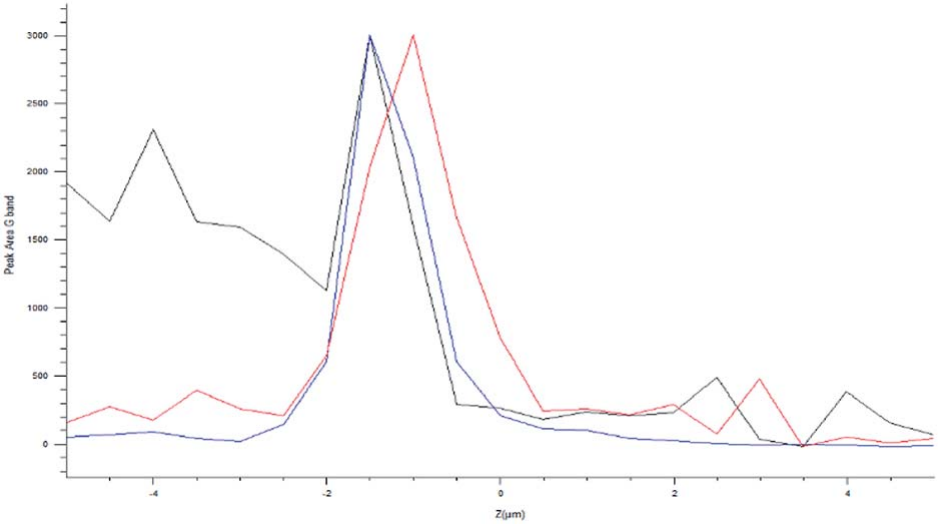
Raman point series in *Z* axis.

The optical images reconstructed by means of spectroscopic data (Fig. 13) allow for identifying the presence of nanotube in the cell on the *XY* plane. In Fig. 12c and d, it is visible the specific localization of the was MWCNT carrier (bright spot, c panel) in the cell. To verify whether it inside or on the surface of the cell, a series of measurements was carried out in *Z*-axis, at step of 0.5 μm, setting as the 0 of the *Z*-axis the chosen focus point, which coincides with the cell surface (Fig. 14). Being the nanotube below 0 it is possible to say that the same is under the focus point, therefore under the cell surface, a sure indication that the nanovector was internalized by the cell. This statement is confirmed by the precision of the instrument being equal to 1 μm.

Unfortunately, the nanosphere and nanoaggregate samples, have too low Raman signal to distinguish it from the biological signals of the cell (results for these samples have not been reported).

### 3.6 Aerosol deposition characterization

**Fig. 15:**
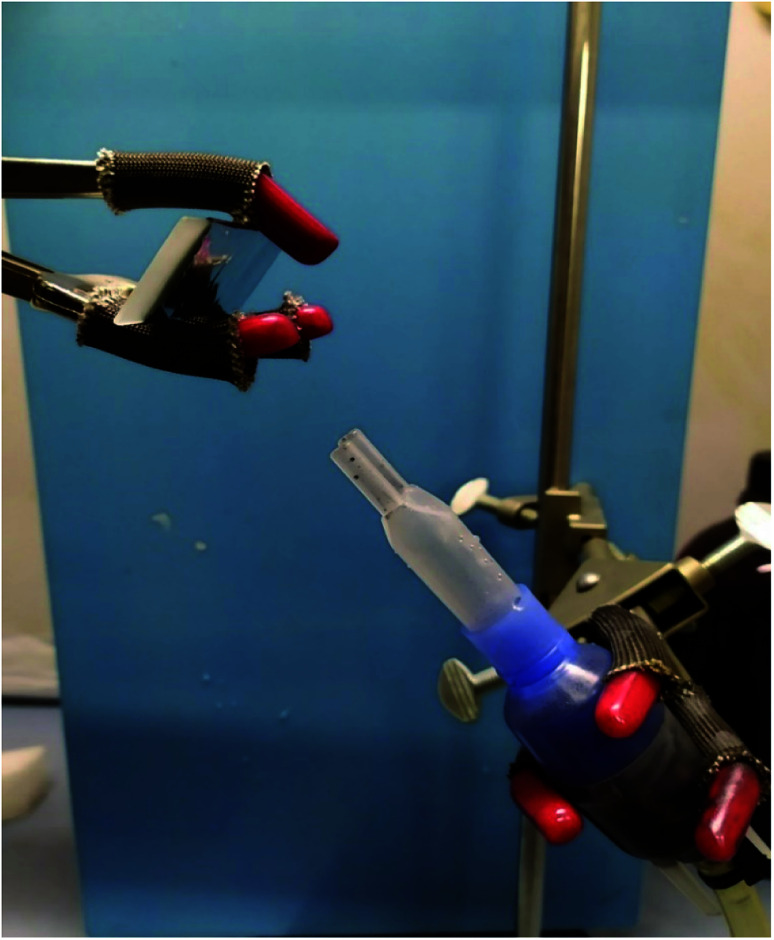
The set-up used for the deposition.

**Fig. 16:**
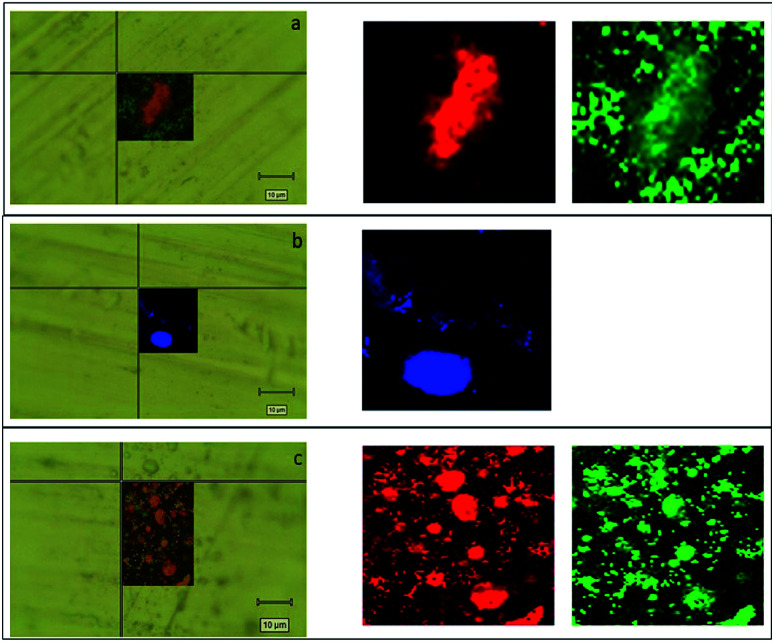
(a) Mapping of MWCNT-COOH/Alg 10 : 1 sample obtained on the droplet by Raman investigation overlapped at the optical image. The area of the D peak of MWCNT is reported in red and the area of alginate peak is reported in green. (b) Mapping of alginate nanoaggregates sample obtained on the droplet by Raman investigation overlapped at the optical image. The blue map represents the area employed by alginate. (c) Mapping of alginate nanospheres sample obtained on the droplet by Raman investigation overlapped at the optical. The red and green maps report the area occupied by nanospheres.

To our best knowledge, the Raman imaging has been carried out to characterize the internal structures of atmospheric aerosol:^56,57^ we chose to transfer, for the first time, this technique for studying the structure of aerosol particulates obtained by alginate nanoparticles and MWCNT-COOH/Alg complexes (Fig. 15). It is known that aerosol administration is a powerful tool to deliver efficiently drug in the nose.^32,58–61^ However, aerosol particulate must have peculiar features. In order to characterize these features, we carried out aerosol deposition tests for all drug delivery systems. The aerosol deposition tests have shown interesting results, as for almost all samples the phenomena of aggregation are reduced. For the MWCNT/Alg 10 : 1 sample in Fig. 16a, we can see how the droplets with an approximate diameter of 10 μm are isolated and the aggregation phenomena in the drop is reduced. The Raman mapping was performed on the D-band (red) of the carbon nanotubes, due to superimposition of G band and alginate peak at around 1600 cm^−1^, that shows how the nanotube is positioned inside the droplet, while the green mapping is relative to the vibration signal of the C–H at 2950 cm^−1^ related to alginate. In Fig. 16b, which shows the nano-aggregated sample of alginate, one can see single systems such as submicrometric diameter dots, where the mapping was carried out on the band at 2950 cm^−1^. For the nanospheres, the Fig. 16c shows the nanometric droplets with the mapping carried out on the signals at 2950 cm^−1^ (red) and 1424 cm^−1^ of the vibrations of the C–O bonds of carboxylic acids.

In conclusion, it is possible to say that for all the treated samples the phenomena of aggregation were reduced. These results demonstrated that nanocarriers are suitable for a possible nose to brain application: in fact, an aerosol particulate, having small droplets size and containing no aggregate carriers inside, could reach undamaged the olfactory epithelium and fulfil their functions in the foreseen site.

## 4 Conclusions

In order to improve the scientific knowledge, alginate nanoparticles, as an alternative to the classic chitosan ones, were obtained using different synthetic techniques. The reactions were optimized to find the best ratios between the reagents. These vectors were engineered for the paracellular route. Then, using the same biopolymer, MWCNT-COOH based nanovectors were generated, and the most stable MWCNT-COOH/alginate 10 : 1 was selected, engineered to travel the axonal or transcellular paths because of the structural and chemical–physical characteristics.

All nanovectors were characterized by SEM, Raman and ATR-FTIR spectroscopy. The drug loading occurs both on alginate nanovectors and MWCNT-COOH based ones. Afterwards, the release was evaluated, obtaining a slow release (36 h) for the alginate nanoaggregate sample. The toxicity was evaluated on the SHSY-5Y cell line, yielding the result that all samples showed non-toxic characteristics. In particular, the cells treated with the samples in the absence of MWCNT-COOH, showed a slight proliferative behaviour. The internalization of MWCNT-COOH/Alg was demonstrated by means of Raman spectroscopy on the SHSY-5Y cell line. Finally, a method of self-administration, such as aerosol, was studied: the results show the formation of small droplets, fine particulate matter and limited aggregation, further studies, which will be carried out in the near future, concern the testing of the system in animal models.

## Conflicts of interest

There are no conflicts to be declared.

## Supplementary Material

RA-010-D0RA04212A-s001
