# Supplementary material for: Novel optimized biopolymer-based nanoparticles for nose-to-brain delivery in the treatment of depressive diseases
Source: RSC Adv. 2020 Aug 4;10(48):28941–9. doi: 10.1039/d0ra04212a (PMC9055835; doi:10.1039/d0ra04212a)
Supplement: RA-010-D0RA04212A-s001 [file RA-010-D0RA04212A-s001.pdf]

Electronic Supplementary Material (ESI) for RSC Advances.
This journal is © The Royal Society of Chemistry 2020

SUPPLEMENTARY MATERIAL

SEM images of the lowest Ca^{2+} concentration test (1S), the dilution test from synthesis obtained by ARV-310 (2s) and the qualitative dispersion test (3S) are reported.

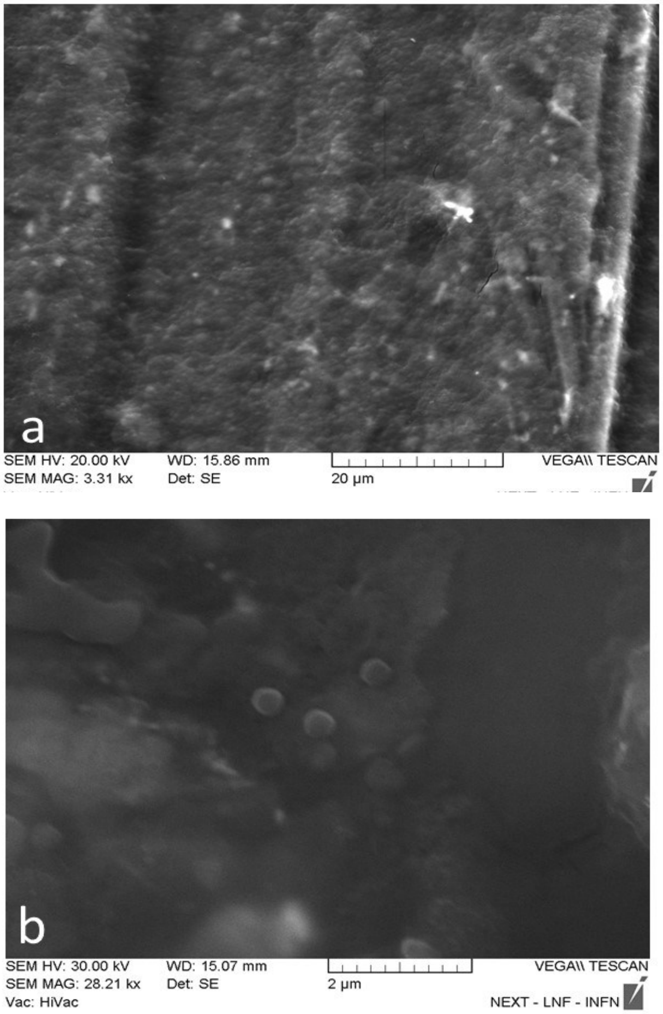

Figure 1S in (a) lower calcium concentration (ratio 3:1 alginate:Ca2+) at a surfactant concentration of 5 10-3 M and in (b) 1:1000 dilution of (a).

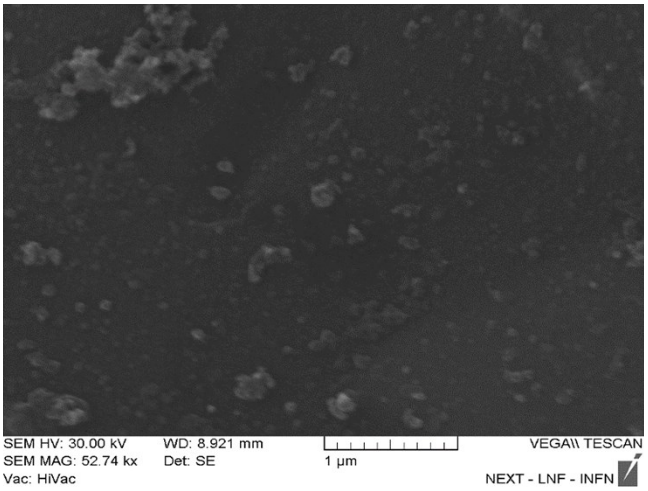

Figure2S Dilution test 1:1000 of synthesis using planetary mixer setup (Figure 6(c)).

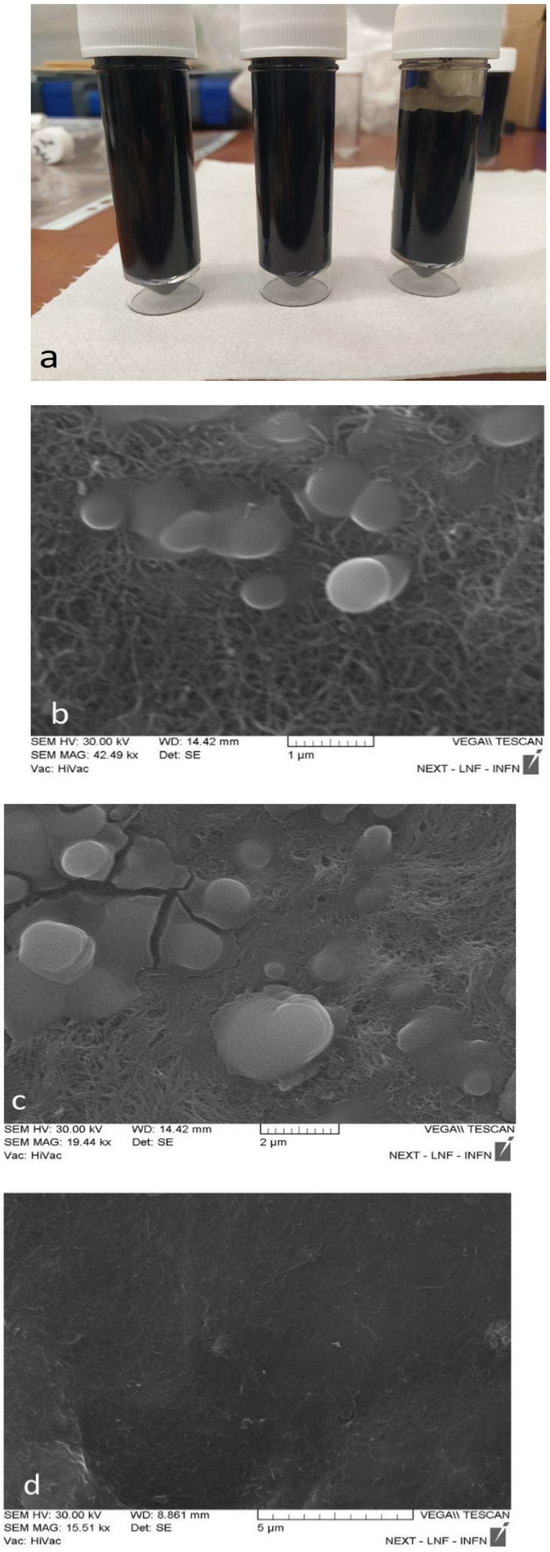

Figure 3S a) Kinetic stability of solutions at 30 days. of samples having from left 10:1, 1:1 and 1:10 ratio, respectively; b) SEM image of sample having 10:1 ratio. c) SEM image of sample having 1:1 ratio; d) SEM image of sample having 1:10 ratio.
